# Comment on: Efficacy of Platelet‐Rich Plasma Therapy in Melasma Using Microinjections and Microneedling Techniques

**DOI:** 10.1111/jocd.70754

**Published:** 2026-02-22

**Authors:** Dandan Wang, Ying Chen, Zhicun Han

**Affiliations:** ^1^ Department of Acupuncture The NO. 4 People's Hospital of Yuyao Yuyao China; ^2^ Department of Acupuncture The Second People's Hospital of Tongxiang Tongxiang China; ^3^ Department of Acupuncture Tongde Hospital of Zhejiang Province Affiliated to Zhejiang Chinese Medical University (Tongde Hospital of Zhejiang Province) Hangzhou China

To the Editor,

1

We read with great interest the article by Sitaula et al. entitled “Efficacy of Platelet‐Rich Plasma Therapy in Melasma Using Microinjections and Microneedling Techniques” [1]. The authors are to be commended for conducting a rigorous randomized split‐face study that addresses a critical clinical question: the optimal delivery modality for platelet‐rich plasma (PRP) in melasma management. Their findings, demonstrating a statistically superior efficacy of microneedling over microinjection (70.21% vs. 36.6% improvement in modified Melasma Area and Severity Index [MASI] score, *p* < 0.001), provide valuable, high‐level evidence that will inform therapeutic decision‐making. The split‐face design effectively mitigates inter‐individual variability, enhancing the validity of the conclusions [2].

While the study robustly establishes the superiority of microneedling, we wish to explore potential mechanistic underpinnings and future research directions to build upon this foundation. First, the authors attribute the enhanced efficacy of microneedling to improved drug penetration via microchannels. This physical explanation is plausible, but we hypothesize that the mechanism may involve active biological synergy. Microneedling induces a controlled wound‐healing response, triggering the release of cytokines and growth factors that modulate the local microenvironment [3]. Concurrently, PRP delivers a concentrated bolus of growth factors (e.g., TGF‐β, PDGF, VEGF), which may synergize with the microneedling‐induced response to amplify regenerative and anti‐melanogenic effects [4]. For instance, network meta‐analyses have highlighted the efficacy of intradermal PRP, potentially mediated by TGF‐β1‐induced inhibition of melanogenesis [5]. This synergy—where physical intervention primes the tissue for enhanced biological response—could explain the significant outcome disparity. Future studies incorporating sequential skin biopsies for immunohistochemical analysis of melanocyte activity (Melan‐A), angiogenesis (CD31), and collagen remodeling pre‐ and post‐treatment could directly test this hypothesis and provide unprecedented mechanistic insight into the combination therapy [6]. However, microinjection combines two types of stimulation: physical and pharmacological. Physical stimulation is initiated by skin puncture, triggering a complex wound healing cascade. Pharmacological stimulation relates to the biological activity of the injected substances. Due to the precise placement of products within the dermis, high concentrations of active ingredients can directly target cells, such as fibroblasts or melanocytes. In contrast, microneedle therapy primarily provides physical stimulation and lacks the ability to directly deliver products to target cells. Therefore, further research is needed to validate the superiority of microneedle technology over microinjection and to explore its underlying mechanisms.

Second, the impressive > 75% improvement in 46.7% of the microneedling group underscores the need for long‐term follow‐up. Melasma is notoriously chronic and recurrent, and durability of treatment response is paramount [5]. While the open‐label design may introduce bias in subjective endpoints, the concordance between modified MASI scores and global assessments strengthens the findings. However, data on relapse rates and duration of response at 6–12 months post‐treatment would clarify whether microneedling with PRP offers transient improvement or sustained disease modification. Such follow‐up would align with emerging research on microneedling‐based strategies for long‐term skin remodeling and controlled drug delivery [6].

In conclusion, we applaud Sitaula et al. for their valuable contribution [1]. Their work definitively establishes microneedling as the superior PRP delivery method for melasma. We encourage further research to unravel the mechanistic synergy and evaluate long‐term outcomes, which could pave the way for optimized combination therapies for this challenging condition.

## Author Contributions

Zhicun Han conceived the idea for this correspondence and drafted the manuscript. Dandan Wang and Ying Chen wrote the manuscript together. All authors reviewed and approved the final version.

## Conflicts of Interest

The authors declare no conflicts of interest.

## Linked Articles

This article is linked to Sitaula et al. papers. To view this article, visit https://doi.org/10.1111/jocd.70246.

## Data Availability

The data that support the findings of this study are available from the corresponding author upon reasonable request.
